# Education

**Published:** 1874-07

**Authors:** W. H. Atkinson


### EDUCATION.

BY W. H. ATKINSON M. D., D. D. S.

*Read before the District Dental Society of the Seventh Judicial District of the State of New York, Rochester, N. Y. June 2d,* 1874.

The education of dentists is a subject that has many aspects of preparation, for the exercise of the functions belonging to a very complicated calling; that involvs a large range of ability both natural and acquired.

Dentistry is clearly a department of medicine and surgery; therefore involves competent instructions and attainments in the principles of these with the additions of special application of the laws and principles of mechanics so necessary to the care preservation and restoration of the masticatory apparatus.

Looking upon what has as been said as necessary preparation to proper qualifications for practice; where shall we find the truly qualified practitioner of dentistry!

Were we to demand each particular operater to know the the range of science already acknowledged as the only legitimate basis of enlightened practice we should not find a single example of qualifications for all the duties rightfully belonging to Dentistry.

But this is no more true of dentistry as a specialty of the healing art than of any other depatment if we take all things into account.

In so far as dentisty is more open, plain and demonstrable, compared to general medicine and surgery or those branches that deal with the more occult organs and functions of body and mind: first to that degree are we bound to be competent in ratio above those more ambiguous and ill defined departments of our great art.

The progress that our specialty has made in the last fifty years is not equalled by any other branch, not excepting ophthalmology and the understanding and care of the apparatus; great and striking as have been the discoveries and inventions in these departments of late; owing principally to the discovery, invention and use of optical and other means of improved observation. In fact, the progress has been so rapid in dentistry that each decade, if not half decade, has added new requirements of qualification so distinctive as to almost forbid the possibility of setting bound to a curriculum of studies that should not be itself a hindrance rather than a help to the beginners in the preparations for acceptable practice.

Whenever this subject is up for consideration, we hear a great variety of views expressed as necessary requirements for private pupilage, the self-sufficient leaders who never went to college of any sort classical or professional, but pick up their knowledge "by hook and by crook" in or out of the office of some one already in practice, are loudest in denouncing the short-comings of the "Teachers" in colleges, the majority of whom could give better "reasons for the hope that is in them" than these earnest and useful "practical" members of our advancing body!

In so saying I do not mean to endorse the "errors" of the schools or to intimate that they are above improvement. But I do wish most emphatically to affirm that we are better off as a profession by reason of their existence, lame and impotent as they confessedly are, than we would be without them, even as they have been. But when we reflect that they too are in earnest to make an improved record in the coming time, I rejoice in their existence no less than in the strictures that are so freely and sometimes ruthlessly and inconsiderately passed upon them. Let one ask those who so severely denounce the "manufacture" of dentists in the schools, "my dear brethren what have you done to improve the quality of the coming members of our body?

Have you freely spent valuable time in instructing the 'boys' near you, in your very best and latest and most valued attainments? upon which perch as the distinguishing characteristics of your own 'differencc' from the 'spume of the colleges' sent broadcast into the community to learn dentistry by the same old method of 'doing wrong to learn how to do right?"

Be assured beloved co-laborers in an arduous work, one and all, the great incubus upon the proper education of the dentist is, the prevailing animus of teaching, learning and practicing our science and art for the acquisition of money, rather than the fulfilment of a redemptive mission in teaching, learning and practicing how to avoid the dire necessities that called dentistry into being!

On the other hand I could hardly desire that there was a law against graduating anybody in our profession. For I verily believe that the possession of a diploma is a disadvantage to all who care for it or value it.

For it tempts them to account themselves as having "attained" at least the approval of men high in station and may be in merit and knowledge also!

I hold as a great hindrance to our advancmcent the desire to be "recognized" "acknowledged" as a "learned member of a learned body" above the desire to be learned—able and competent to grapple with the enemy in his stronghold, even though it be a lonesome and obscure yet successful struggle. What proportion of your acquaintances practice the study and execution of difficult work for the love of it?

And what proportion pursue their calling for the pay in the sense of gain?

The replies to these queries will settle the question what hinders the true progress of dental education.

A man's standing among his fellow men may be determined by a vote; but his knowledge can never be the thing effected. for this is dependent upon the facts of knowing or not knowing which constitutes his endowment.

So, if any one says he can show a better way to teach or practice any plan or operation, let us treat all such politely and give them opportunity to show us "the more excellent way."

If it were the last time I had to speak upon the subject to my beloved brethren, old and young, I would repeat the old saying as pre-eminently pertinent and successful whenever carried but in the sincerity of the heart: "Let brotherly love continue!" And again have no contentions among you; but rather emulations, in which each shall strive to show forth who can best work and who can best agree "in searching out the deep truths that lay at the bottom of the principles of the science that crop out in the physiology, pathology, therapeutics, prophylasis and prosthetics; and applying them to practice under the open frankness of plainness of understanding and freedom of communication to me and to all, that the truth may be confirmed in the mind of each and for the good of all.

